# TLR5 mediates CD172α^+^ intestinal lamina propria dendritic cell induction of Th17 cells

**DOI:** 10.1038/srep22040

**Published:** 2016-02-24

**Authors:** Han Liu, Feidi Chen, Wei Wu, Anthony T Cao, Xiaochang Xue, Suxia Yao, Heather L Evans-Marin, Yan-Qing Li, Yingzi Cong

**Affiliations:** 1 Department of Gastroenterology, The Qilu Hospital, Shandong University, Shandong, China; 2 Department of Microbiology and Immunology, and University of Texas Medical Branch, USA; 3 Department of Pathology, University of Texas Medical Branch, USA; 4 Department of Gastroenterology, The Shanghai Tenth People’s Hospital, Tongji University, Shanghai, China; 5 Department of Biopharmaceutics, School of Pharmacy, Fourth Military Medical University, Xi’an, China

## Abstract

Multiple mechanisms exist in regulation of host responses to massive challenges from microbiota to maintain immune homeostasis in the intestines. Among these is the enriched Th17 cells in the intestines, which regulates intestinal homeostasis through induction of antimicrobial peptides and secretory IgA among others. However, the means by which Th17 cells develop in response to microbiota is still not completely understood. Although both TLR5 and CD172α^+^ lamina propria dendritic cells (LPDC) have been shown to promote Th17 cell development, it is still unclear whether TLR5 mediates the CD172α^+^LPDC induction of Th17 cells. By using a microbiota antigen-specific T cell reporter mouse system, we demonstrated that microbiota antigen-specific T cells developed into Th17 cells in the intestinal LP, but not in the spleen when transferred into TCRβxδ^−/−^ mice. LPDCs expressed high levels of TLR5, and most CD172α^+^LPDCs also co-expressed TLR5. LPDCs produced high levels of IL-23, IL-6 and TGFβ when stimulated with commensal flagellin and promoted Th17 cell development when cultured with full-length CBir1 flagellin but not CBir1 peptide. Wild-type CD172α^+^, but not CD172α^−^, LPDCs induced Th17 cells, whereas TLR5-deficient LPDC did not induce Th17 cells. Our data thereby demonstrated that TLR5 mediates CD172α^+^LPDC induction of Th17 cells in the intestines.

The intestinal tract is exposed to massive amounts of foreign antigen stimuli, including commensal bacteria, pathogens, and dietary components. Multiple mechanisms have been developed in the regulation of host responses to such stimulation to maintain immune homeostasis in the intestines^1^,^2^. The intestinal mucosal surface is a natural site for the development of Th17 cells, which produce a distinct set of cytokines, including IL-17A (IL-17), IL-17F, IL-21 and IL-22. It has been shown that intestinal Th17 cell development is stimulated by a specific species of microbiota^3^, with segmented filamentous bacteria (SFB) being identified as one of such stimulators^4^. Although both pro- and anti-inflammatory functions of Th17 cells have been demonstrated in different experimental systems^5^,^6^,^7^,^8^, the enrichment of Th17 cells in the intestines suggests a role for these cells in mucosal homeostasis and more specifically in the containment of the vast local microbiota. IL-17, a signature Th17 cell cytokine, is able to stimulate intestinal epithelial cell production of antimicrobial peptides^9^,^10^. We and others have shown recently that Th17 cells induce mucosal IgA production in the intestines and lungs^11^,^12^,^13^, both of which could contribute to the maintenance of intestinal homeostasis. However, the cells and environmental factors which promote Th17 cell development in intestines are still not completely understood.

Intestinal dendritic cells (DCs) form an extensive network in lamina propria and are critical in both shaping innate and adaptive immune responses to commensal microbiota, as well as instructing lymphocytes homing to the intestines, by inducing the expression of the gut-homing receptors α_4_β_7_ and CCR9 on these cells. Thus DCs play a key role in controlling intestinal inflammation and maintenance of immune homeostasis^14^,^15^,^16^,^17^. Multiple, phenotypically distinct subsets of DCs exist in intestinal lamina propria, including CD11c^+^CD11b^+^CD8α^−^ (mDC), CD11c^+^CD11b^−^CD8α^+^ (lymphoid DC), and CD11c^+^CD11b^−^B220^+^ (pDC)^14^,^18^. Some small intestinal lamina propria DCs express CX_3_CR1, and CX_3_CR1^+^ DCs may serve as a gateway for the uptake of microbiota by the continuous sampling of luminal content via transepithelial dendrites in certain sites within the intestines^19^,^20^. More recently, it has been shown that subsets of LPDCs express CD103, and that CD103^+^ and CD103^−^ LPDCs play different roles in generating gut-trophic T cells and in inducing B cell IgA production, thus regulating T cell and B cell responses^21^,^22^. Four subsets of DCs can be identified based on CD103 and CD11b expression in the intestines^18^; however, the specific role of most of these DC subsets in enteric bacterial antigen sampling and presentation is unknown. Moreover, it also remains unclear whether these distinct DC subsets work synergistically or have distinct functions in response to intestinal microbiota, and thus in instructing adaptive immune responses and the differentiation of different types of effector T cells. Although a series of studies show an impressive degree of flexibility or “plasticity” of DCs in response to different microbial stimuli^23^, accumulating evidence suggests that distinct DC subpopulations may have intrinsic biases in their capacities to process and present antigens, thus stimulating qualitatively different types of immune response^24^,^25^,^26^. The various DC subsets have been shown to differentially express TLRs, as well as to respond differently to microbial stimuli^27^. For example, while CD8α^−^ DCs have an overall higher phagocytic capacity, CD8α^+^ DCs internalize apoptotic cells^28^. In response to stimulation by TLR 3, 4, 7, and 9, only myeloid DC (mDC), but not plasmacytoid DC (pDC), produce IL-23, although both mDC and pDC produce IL-12^29^. Although it has been shown that generation of Th17 cells in intestinal mucosa requires bacterial antigens^30^,^31^, it is still not completely understood what subsets of LPDC present the bacterial antigen to induce Th17 cell development. CD103^+^CD11b^+^ DCs, which express TLR5 and are considered to exert innate function through detecting flagellin via activation of TLR5 signaling^32^,^33^, were shown recently to be able to promote intestinal Th17 cell development through production of IL-6^34^,^35^. TLR5 has also been implicated in LPDC induction of Th17 cells in intestines. Signal regulatory protein alpha (SIRPα/CD172α) is a conserved transmembrane protein^36^. CD172α^+^ LPDCs are able to promote Th17 cell development^34^. However, it is still unclear whether TLR5 mediates CD172α^+^ LPDC induction of Th17 cells in the intestine in response to commensal bacteria, and thus controls immune homeostasis and intestinal inflammation. We report here by using a microbiota antigen-specific T cell reporter system that LPDCs express high levels of TLR5. Most TLR5^+^ LPDCs also co-express CD172α. Additionally, CD172α^+^ but not CD172α^−^ LPDCs induce microbiota antigen-specific Th17 cells, which is mediated by TLR5.

## Results

### Different cytokine profiles of CBir1 Tg CD4 T cells in spleen and lamina propria of recipients upon mucosal challenge with CBir1 flagellin

**Figure 1:**
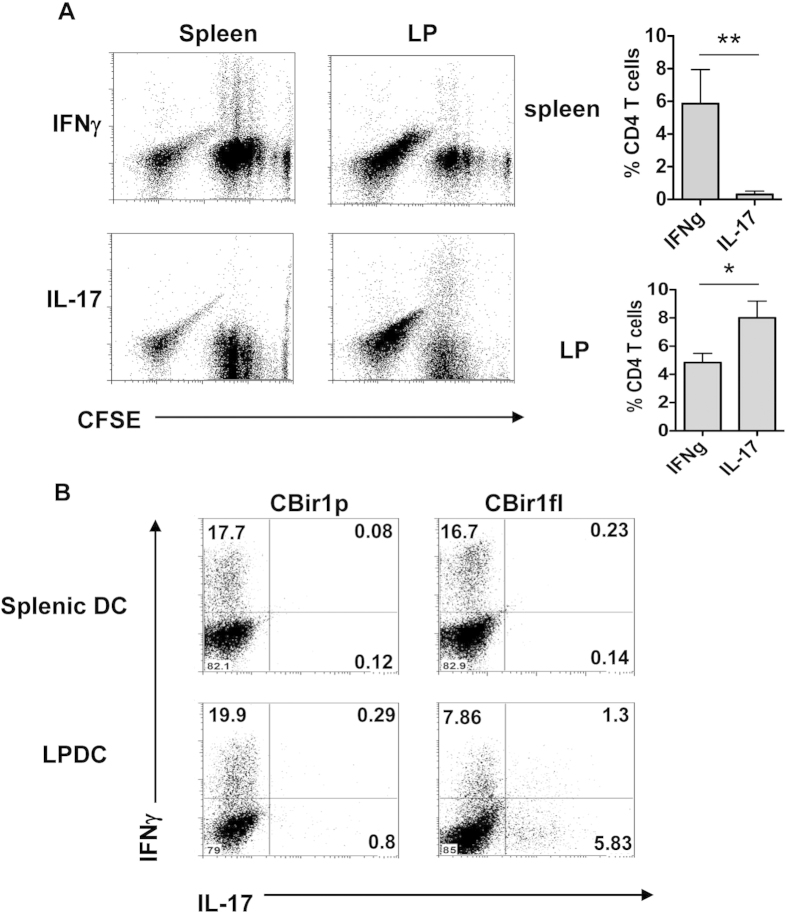
Intestinal LPDCs induce Th17 cell development. (**A**) CBir1 Tg CD4 T cells were labeled with CFSE and transferred into TCRβ×δ^−/−^ mice. The mice were gavaged with CBir1 flagellin the same day. Cytokine production of CBir1 Tg CD4 T cells in spleen and intestinal LP was determined by intracellular staining 3 days later. FACS plots are representative of 3 independent experiments. Bar charts of cytokine expression from pool of 3 experiments. *p<0.05, and **p<0.01. (**B**) CBir1 Tg CD4 T cells were cultured with splenic DC or LPDC isolated from C57BL/6 mice in the presence of full-length CBir1 flagellin or CBir1 peptide for 5 days. Cytokine production of CBir1 Tg CD4 T cells was determined by intracellular staining. FACS plots are representative of 3 independent experiments.

We have previously demonstrated that CBir1 TCR transgenic (Tg) T cells, which are specific to an immunodominat microbiota antigen CBir1 flagellin, do not respond to CBir1 flagellin in the intestinal lumen, as secretory IgA blocks the translocation of commensal bacteria and their antigens across the intestinal epithelium in wild-type mice^37^. To investigate the T cell response to microbiota antigen, we transferred CFSE-labeled CD4^+^ T cells from CBir1 Tg mice into TCRβ×δ^−/−^ mice, in which the intestinal IgA was dramatic decreased, and CBir1 flagellin was able to translocate cross the epithelium^11^. The recipient mice were gavaged with recombinant CBir1 flagellin. Three days after oral CBir1 challenge, spleen cells and intestinal lamina propria lymphocytes were isolated from recipients and re-stimulated with PMA/Ionomycin for 5 hrs. Intracellular IFNγ and IL-17 were measured. CBir1 Tg T cells produced IFNγ, but not IL-17, in the spleen, whereas lamina propria T cells produced both IFNγ and IL-17 (Fig. 1A), indicating that intestinal environment allowed effector CD4^+^ T cell development into both Th1 and Th17 cell pathways, whereas the spleen supported only the Th1 cell pathway, in response to mirobiota stimulation.

As DCs have been shown to instruct T cell development into different subsets, we then investigated whether DC in spleen and intestinal LP differentially induce Th1 and Th17 cell development. We cultured CBir1 T cells with DC isolated from spleen or intestinal LP in the presence of full-length CBir1 flagellin with TLR5 activity or CBir1 peptide without TLR5 activity. Five days late, IFNγ and IL-17 production was measured by flow cytometry. In response to CBir1 peptide, CBir1 T cell only produced IFNγ but not IL-17 when stimulated with both splenic DC and LPDC. Interestingly, in response to full-length CBir1 flagellin, although splenic DC induced CBir1 T cell production of IFNγ but not IL-17, LPDC induced CBir1 T cell production of both IFNγ and IL-17 (Fig. 1B), indicating that spleen DC and LPDC differentially induce Th1 and Th17 cell development in response to stimulation of full-length CBir1 flagellin.

### MLN DC and lamina propria DCs take up CBir1 given orally, and stimulate T cell response

**Figure 2:**
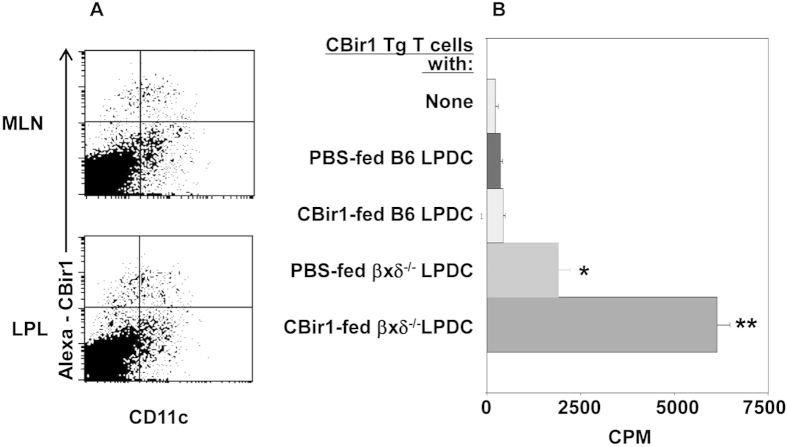
MLN DC and LPDC take up and present CBir1 flagellin to T cells after mucosal challenge *in vivo*. Wild-type and TCRβ×δ^−/−^ mice were gavaged with Alexa 647-labeled CBir1 flagellin. Uptake of CBir1 flagellin by MLN DC and LPDC was determined 2 hrs later by flow cytometry (**A**). When cultured with CD4 T cells of CBir1 Tg mice, LPDC from CBir1-gavaged TCRβxδ KO, but not wild-type, mice stimulated T cell proliferation (**B**). Data are reflective of 3 independent experiments. *p<0.05, and **p<0.01 compared to controls.

To determine whether DCs in MLN and lamina propria can take up CBir1 flagellin given mucosally, wild type or TCRβ×δ^−/−^ mice were given Alexa 647-labeled CBir1 by gavage. Alexa 647-labeled CBir1 flagellin behaved in a physiological manner in that Alexa-CBir1 flagellin stimulated CBir1 Tg T cell proliferation at a level similar to that of unlabeled CBir1 flagellin, and the cytokine profiles were also similar (data not shown). Two hrs later, MLN DC and LPDC were isolated, and the uptake of CBir1 by DC was determined by flow cytometry. Subsets of both MLN DC and LPDC were Alexa-647^+^ in TCRβ×δ^−/−^, but not wild type mice, indicating CBir1 uptake by these DCs (Fig. 2A). These CBir1-bearing LPDCs of TCRβ×δ^−/−^ mice stimulated CBir1-specific T cell proliferation (Fig. 2B), but did not stimulate OVA-specific T cells (data not shown) when cultured with CD4 T cells from CBir1-specific Tg mice or OVA-specific OT II mice, demonstrating that LPDC can present this commensal bacterial antigen to T cells after *in vivo*exposure. LPDC from CBir1-fed, wild-type B6 mice did not stimulate CBir1 T cell proliferation (data not shown). Interestingly, LPDC from PBS-gavaged TCRβ×δ^−/−^ mice also stimulated CBir1 Tg T cell proliferation, but to a much lower degree, indicating that endogenous CBir1 flagellin in the intestinal lumen crosses the mucosal barrier and is taken up by LPDC. LPDC from PBS-gavaged mice did not stimulate OVA-specific OT II T cell proliferation (data not shown).

### LPDC expression of TLR5 and CD172α

**Figure 3:**
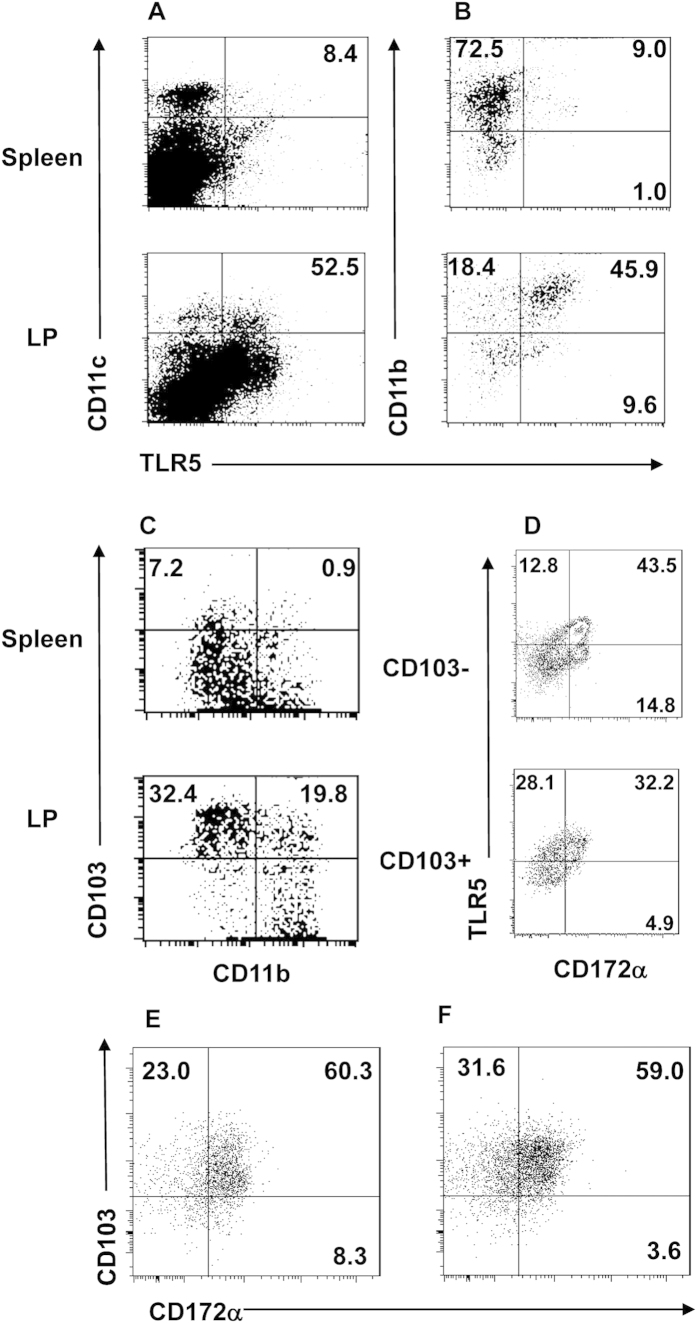
More LPDCs express TLR5 than spleen DCs and most CD172α^+^ LPDCs are also TLR5^+^. Spleen cells and LP cells were isolated from C57BL/6 mice. After depleting CD4^+^ T cells, CD4^−^ cells were stained with CD11c, CD11b, CD103, and TLR5 and analyzed by flow cytometry. (**A**) CD11c^+^ cell expression of TLR5 was shown. (**B**) TLR5 expression by CD11b^+^ and CD11b^−^ DCs by gating on CD11c^+^ population. (**C**) CD11c^+^ DC expression of CD103 and CD11b by gating on CD11c^+^ population. (**D**) Expression of TLR5 and CD172α by CD103^+^ DC and CD103^−^ DCs in LP of wild-type C57BL/6 mice by gating on CD11c^+^CD11b^+^ population. (**E,F**) CD172α expression by CD103^+^ DC and CD103^−^ DCs in LP of wild-type (**E**) and TLR5 KO C57BL/6 mice (**F**) by gating on CD11c^+^ population. FACS plots are reflective of 2–4 independent experiments.

Multiple subsets of DC exist in different tissues. CD11c^+^CD11b^+^CD8α^−^ (mDC), CD11c^+^CD11b^−^CD8α^+^ (lymphoid DC), and CD11c^+^CD11b^−^B220^+^ (pDC) have been identified in the intestinal lamina propria^18^. DCs are known to express a variety of TLRs. Because TLR5 is the receptor for flagellin, we determined TLR5 expression in the spleen and LPDC. Consistent with a previous report^32^,^33^, compared to spleen DC, more CD11c^+^LPDC expressed TLR5 (Fig. 3A). Both CD11c^+^CD11b^+^ and CD11c^+^CD11b^−^ DC expressed TLR5, but more CD11c^+^CD11b^+^ LPDC expressed TLR5 than did CD11c^+^CD11b^−^ LPDC (Fig. 3B). These results are similar to those using Alexa-CBir1 flagellin as a TLR5 ligand (data not shown), confirming that Alexa-CBir1 flagellin does bind TLR5 specifically.

It has been shown that CD103^+^DC are a subset of mucosal DC instructing T and B cells to become gut-trophic and migrate into the intestines^21^. CD103^+^ DCs play a role in regulating a mucosal T cell response, whereas CD103^−^ DCs stimulate mainly effector T cell responses^21^. More LPDC are CD103^+^ than are spleen DC (Fig. 3C). Both CD103^+^ and CD103^−^ DC expressed CD172α^+^ at similar levels (47.6% VS. 43%). Interestingly, a majority of CD172α^+^ DCs in the intestines also expressed TLR5 in both CD103^+^ and CD103^−^ DCs (Fig. 3D). Deficiency in TLR5 did not affect intestinal LP CD172α DC population (Fig. 3E,F).

### CBir1 flagellin stimulation results in differing cytokine production profile and antigen-presenting ability between LPDC and spleen DC

**Figure 4:**
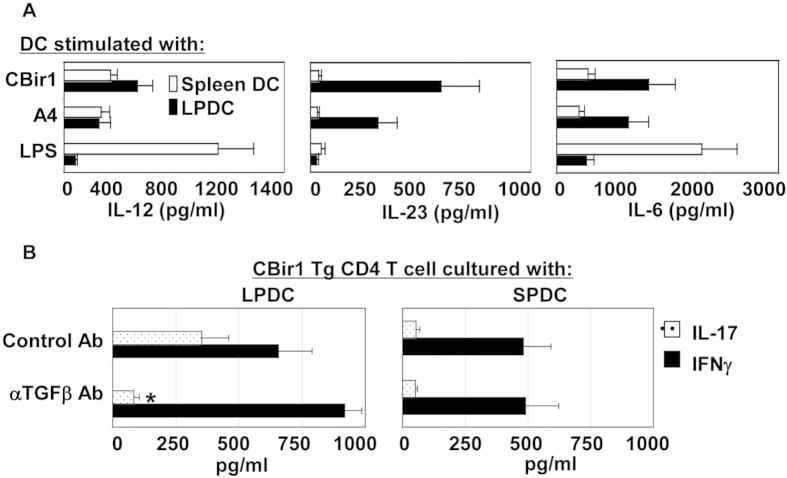
Different cytokine profiles and antigen-presenting ability of spleen DC and LPDC in response to CBir1 flagellin stimulation. (**A**) Spleen DC and LPDC isolated from C57BL/6 mice were stimulated with full-length CBir1 flagellin, A4 bacteria, and LPS. IL-12, IL-23, and IL-6 production in the supernatant was measured by an ELISA 24 hrs later. (**B**) CBir1 Tg T cells were cultured with spleen DC or LPDC with or without anti-TGFβ. Cytokine production in the supernatant was determined by an ELISA 3 days later. Data are reflective of 4 independent experiments. *p<0.05, **p<0.01.

Flagellins can activate innate immune cells via TLR5. To determine whether the differing TLR5 expression in spleen DC and LPDC affected their response to CBir1 flagellin stimulation, spleen DC and LPDC were isolated from C57BL/6 mice and stimulated with full-length CBir1 flagellin or flagellated A4 bacteria which produce CBir1 flagellin. Because it has been reported that expression of TLR4 is much lower in LPDC than in spleen DC^33^, lipopolysacchirde (LPS) was also used to stimulate spleen DC and LPDC as a control. Cytokine production was measured by an ELISA in supernatant from a 24-hr culture. As shown in Fig. 4A, spleen DC produced high levels of IL-12p70 and IL-6, and low levels of IL-23 in response to LPS. However, they produced much lower amounts of IL-12p70 and IL-6 upon stimulation with CBir1 flagellin or flagellated A4 bacteria. In contrast, LPDC responded poorly to LPS stimulation, but produced high levels of IL-12p70, IL-23 and IL-6 in response to CBir1 flagellin or flagellated A4 bacteria. To determine whether these differing cytokine profiles for spleen DC and LPDC would induce different T cell responses when presented with CBir1 flagellin, we cultured CD4 T cells from CBir1 Tg mice with spleen DC and LPDC from C57BL/6 mice in the presence of CBir1 flagellin, and measured cytokine production in the supernatant of a three-day culture. CBir1 Tg T cells produced both IL-17 and IFNγ in the presence of LPDC, whereas they produced only IFNγ with spleen DC. Addition of anti-TGFβ antibody inhibited CBir1 Tg T cell IL-17, but increased IFNγ production of T cells with LPDC, but not by spleen DC (Fig. 4B), indicating that in response to enteric flagellin stimulation, spleen DC and LPDC produce a different cytokine environment that may instruct different pathways of effector T cell development. These data also suggest that upon CBir1 flagellin stimulation, LPDC, but not spleen DC, produce or have acquired TGFβ which contributes to stimulation of Th17 cell development in the intestines. This might be a unique feature of LPDC important for driving Th17 cell development in lamina propria.

### TLR5^+^ Lamina propria CD172α^+^ DC induces Th17 cells

**Figure 5:**
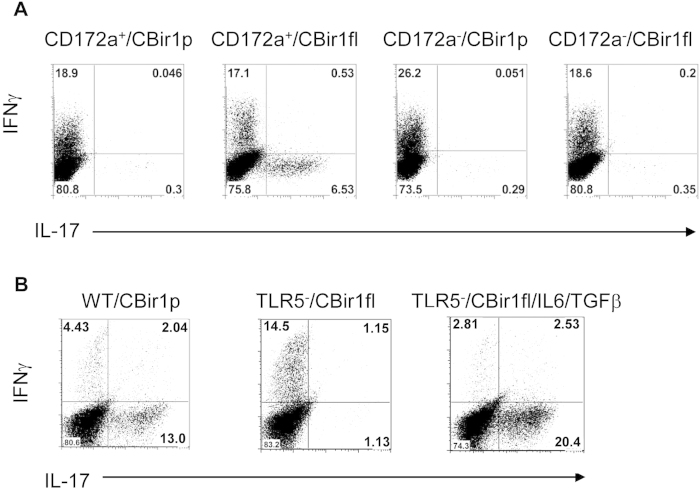
Wild-type, but not TLR5-deficient, CD172α^+^ LPDCs induce Th17 cell development. (**A**) CD172α^+^ and CD172α^−^ LPDCs were isolated from C57BL/6 mice and cultured with CBir1 Tg T cells in the presence of full-length CBir1 flagellin or CBir1peptide. Cytokine production of CBir1 T cells was determined by intracellular staining 5 days later. FACS plots are reflective of 2 independent experiments. (**B**) LPDCs were isolated from wild type or TLR5 KO mice and cultured with CBir1 Tg T cells in the presence of full-length CBir1 flagellin or CBir1peptide. Cytokine production of CBir1 T cells was determined by intracellular staining 5 days later. FACS plots are reflective of 2 independent experiments.

To determine if CD172α^+^ LPDCs are able to promote a microbiota antigen-specific Th17 cell response, we isolated CD172α^+^ and CD172α^−^ LPDCs from intestines of wild-type B6 mice and cultured them with CBir1 Tg T cells in the presence of full-length CBir1 flagellin, which is able to activate TLR5 of DC and CBir1 T cells, or CBir1 peptide, which is able to activate CBir1 T cells but will not activate TLR5 of DC, as it lacking a TLR5-binding site. As shown in Fig. 5A, while both CD172α^+^ and CD172α^−^ LPDCs were able to activate CBir1 T cells to produce IFNγ in the presence either full-length CBir1 flagellin or CBir1 peptide, CD172α^+^, but not CD172α^−^, LPDCs stimulated CBir1 T cell production of IL-17 in the presence of full-length CBir1 flagellin. Moreover, CD172α^+^ LPDCs did not stimulate CBir1 T cell production of IL-17 in the presence of CBir1 peptide. Collectively, these data indicate that CD172α^+^ LPDCs promote Th17 cell development in response to microbiota antigens, which is mediated by TLR5 activation. However, the fact that CD172α^−^ LPDCs do not induce Th17 cell development even in response to stimulation of full-length CBir1 flagellin, which is able to activate TLR5 signaling, indicates that TLR5 signaling alone is not sufficient to promote Th17 cell development.

To further confirm the role of TLR5 in mediating CD172α^+^ LPDCs promotion of Th17 cell development, we isolated LPDCs from TLR5^−/−^ mice and compared them with wild-type LPDCs in their ability to induce CBir1 Tg T cell development into Th17 cells in response to microbiota antigen stimulation. When stimulated with full-length CBir1 flagellin, wild-type LPDCs induced CBir1 Tg T cell production of IL-17, whereas TLR5-deficient LPDCs failed to induce CBir1 Tg T cell production of IL-17 (Fig. 5B). Both wild-type and TLR5 deficient LPDCs were able to induce CBir1 Tg T cell production of IFNγ. Failure of TLR5-deficient LPDC induction of Th17 cell development was not an intrinsic defect, as TLR5-deficient LPDCs were able to induce CBir1 Tg T cell production of IL-17 under Th17 polarization conditions, i.e., in the presence of TGFβ and IL-6 (Fig. 5B). These data further demonstrated that TLR5 mediates CD172α^+^ LPDCs promotion of Th17 cell development in response to microbiota antigen stimulation.

## Discussion

The mucosal surface of the intestines is exposed to a massive number of challenges from microbiota and, occasionally, from pathogens^1^. Among mutiple mechanisms that maintain intestinal homeostasis, T cells have been crucial in preventing chronic intestinal inflammation as well as in maintaining a host immune response to microbiota^38^. However, the means by which a T cell response to microbiota is regulated is still not completely clear. We demonstrated by using a micobiota antigen-specific TCR Tg reporter mice, that DCs instructed T cells to develop into Th17 cells in response to microbiota at the mucosal surface, which was mediated by LPDC production of IL-6, IL-23 and TGFβ. Among multiple subsets of lamina propria DCs, CD172α^+^ DCs were able to induce Th17 cell development, which was mediated by TLR5.

The intestinal tract has been shown as a natural site for the development of Th17 cells. Although both pro- and anti-inflammatory functions of Th17 cells have been demonstrated in different experimental systems, the enrichment of Th17 cells in the intestines suggests a role for these cells in mucosal homeostasis and more specifically in the containment of the vast local microbiota. Great efforts have been made to determine the mechanisms and factors mediating intestinal Th17 cell development and function. It has been shown that the intestinal Th17 cell development is stimulated by specific species of microbiota, with SFB being recently identified as one of such stimulators. SFB colonization of the ileum, and Peyer’s patches in particular, is capable of inducing LPDC production of IL-6 and IL-23^4^,^30^,^31^, the cytokines important in driving Th17 cell development. SFB induction of Th17 cells requires antigen recognition and presentation of SFB antigens requires MHC class II on CD11c^+^ DCs. This process occurs within the LP but independent of secondary lymphatic tissue, which may mean that LPDCs play a crucial role in presenting microbiota antigen to drive Th17 cell development. We have previously identified commensal flagellins as immunodominant microbiota antigens in the intestines^39^. Moreover, flagellin can function as an antigen to induce adaptive T cell response and as a TLR5 ligand to induce innate response. Consistently, CBir1 Tg T cells, which are specific for an immunodominant commensal antigen CBir1 flagellin, developed into Th17 cells in intestines but not in spleens when transferred into TCRβ×δ^−/−^ mice. LPDC, but not splenic DC, expressed high levels of TLR5 and produced IL-23, IL-6 and TGFβ in response to stimulation of CBir1 flagellin but not LPS, and thus could provide a cytokine milieu favoring Th17 cell development, which is consistent with a previous report. Indeed, when cultured with microbiota antigen CBir1-specific Tg T cells, LPDCs induced a Th17 response, whereas splenic DC induced a Th1-dominant response, indicating that LPDC were able to induce Th17 cells *in vitro*. Interestingly, LPDC induced Th17 cell development only in the presence of full-length CBir1 flaggelin, which can bind TLR5, but not CBir1 peptide, which cannot bind TLR5, indicating that LPDC TLR5 signaling is crucial for Th17 cell development in response to microbiota antigen stimulation.

Several subsets of DCs in the intestines have been shown to induce Th17 cell development. The numbers of CD103^+^CD11b^+^ DCs correlate well with the presence of Th17 cells in the intestines^40^, and selective reduction of intestinal CD103^+^CD11b^+^ DCs in mice results in decreased numbers of Th17 cells in the intestines^41^,^42^, indicating a potential role for such subsets of LPDCs in intestinal Th17 cell development, which could be due to decreased production of IL-6 or IL-23, the cytokines promoting Th17 cell development^43^,^44^. It has been further shown that the CD103^+^CD11b^+^ DCs were dependent on IRF4 and instructed Th17 cell development under steady state conditions as well as after enteric pathogen infection^45^,^46^. Interestingly, CD103^+^CD11b^+^ DCs express TLR5, which could be likely responsible for induction of Th17 cells^32^,^33^. A recent report also demonstrated the presence of CCR2-expressing CD103^−^CD11b^+^ DCs in intestinal LP which selectively primed Th17 cells *in vitro*^47^, and CX3CR1^int^ cells as DCs in the intestines that induce Th17 cells and migrate into the inflammatory regions^48^,^49^. Among four defined subsets of LPDCs based on expression of CD103 and CD11b, three of them express CD172α^50^,^51^, and CD172α^+^ LPDCs are able to preferably induce Th17 cells in the intestines. Loss of CD172α signaling in mice results in a selective reduction in the CD103^+^CD11b^+^ subset of DC as well as decreased Th17 cells in the intestines under steady-state conditions and infection with an enteric pathogen^47^. These findings may mean that CD172α^+^ populations could be the LPDC subset inducing Th17 cell responses among the CD103^+^CD11b^+^ subset of LPDCs. Consistently, in our study, FACS sorted CD172α^+^, but not CD172α^−^, LPDC induced Th17 cell differentiation in response to microbiota antigen stimulation, further confirming CD172α^+^ LPDC induction of microbiota antigen-specific Th17 cells. Interestingly, most CD172α^+^ LPDCs also co-expressed TLR5, and TLR5-deficient LPDC did not induce Th17 cell development. However, TLR5 deficiency did not affect development of CD172α^+^ LPDC. Thus, our data further argue that TLR5 signaling mediates induction of Th17 cells by various LPDC subsets, including CD172α^+^ LPDCs.

## Materials and Methods

### Mice

C57BL/6 mice, TCRβ×δ^−/−^ mice and TLR5^−/−^ mice were purchased from Jackson Laboratory. CBir1 Tg mice were housed and maintained in the animal facilities of the University of Texas Medical Branch. All experiments were reviewed and approved by the Institutional Animal Care and Use Committees of the University of Texas Medical Branch. All the methods were carried out in accordance with the approved guidelines.

### Antibodies and reagents

Flow cytometry antibodies anti-mouse CD4-magnet beads were purchased from BD Bioscience. Anti-CD4, anti-IL-17, anti-IFNγ, anti-CD11c, anti-CD11b, anti-CD103, anti-CD172α were purchased from Biolegends. Anti-TLR5 antibody was purchased from NOVUS Biologicals.

### Isolation of CD4^+^ T cells

CD4^+^ T cells were isolated by using anti-mouse CD4-magnet beads (Pharmingen, San Diego, CA) and following the protocols of the supplier, as previously described^11^. Briefly, single cells isolated from spleen were incubated with anti-CD4 beads for 30 min at 4 °C. The cells were then placed on a magnetic column to isolate CD4^+^ T cells.

### Preparation of spleen DC and LPDC

Small and large intestinal segments were prepared from the mice and treated with PBS containing 10% FCS, 20 mM HEPES, 100 U/ml penicillin, 100 μg/ml streptomycin, 1 mM sodium pyruvate, and 10 mM EDTA for 30 min at 37 °C to remove epithelial cells and were washed extensively with PBS. Intestinal segments were digested with 400 Mandl units/ml collagenase D (Roche) and 10 μg/ml DNase I (Roche) in RPMI 1640/10% FCS with continuous stirring at 37 °C for 45–90 min. Cells were spun through a 40%/70% Percoll gradient to enrich for DCs. The obtained cells were incubated with PE-conjugated anti-CD11c after FcR blocking, and isolated by MACS. Purified DCs were then stained with different antibodies, and subsets were sorted by FACSVantage SE (BD Biosciences). The purity of the sorted DCs was routinely >95%.

### Flow cytometric analysis

The FACS was performed as previously described^52^. Briefly, after washing with PBS having 0.1% sodium azide plus 2% heat-inactivated newborn calf serum, the cells were incubated with various conjugated mAbs, washed, and fixed in 1% buffered paraformaldehyde, and quantitated by using an LSRII/Fortessa and FACSDiva software (Becton Dickinson, Mountain View, CA). Further analysis was carried out by using FlowJo. A mAb of the same isotype but irrelevant specificity was used as a negative control.

### Intracellular staining for identification of cytokine phenotype

As described previously^52^, 5 × 10^5^ cells were stimulated for 6 hrs with PMA, and ionomycin and monensin were added for the last 2 hrs of culture. After surface staining for CD4, the cells were fixed and permeablized by using Cytofix/cytoperm solution (BD Pharmingen). IL-17 and IFNγ were stained by using conjugated antibodies (Biolegend).

### T cell culture

CBir1 CD4 T cells were cultured with LPDC or spleen DC in the presence of various antigens. Five days later, the cells were collected for intracellular staining. For polarization of Th17 cells, CBir1 CD4 T cells were cultured with APC in the presence of various antigens and TGFβ (5 ng/ml), IL-6 (20 ng/ml), anti-IFNγ (5 μg/ml) for 5 days.

### Statistical analysis

The data from all experiment**s** were analyzed by using Prism (GraphPad). Statistical significance was determined by using Student’s *t* test with paired tests. The results were considered significant at a P value of less than 0.05.

## Additional Information

**How to cite this article**: Liu, H. *et al*. TLR5 mediates CD172α^+^ intestinal lamina propria dendritic cell induction of Th17 cells. *Sci. Rep.* **6**, 22040; doi: 10.1038/srep22040 (2016).
